# Responses of soil enzyme activities and bacterial community structure to different hydrological regimes during peatland restoration in the Changbai Mountain, northeast China

**DOI:** 10.3389/fmicb.2022.1005657

**Published:** 2022-09-02

**Authors:** Ming Wang, Shangqi Xu, Shengzhong Wang, Cong Chen, Yuting Wang, Lei Liu

**Affiliations:** ^1^Key Laboratory of Geographical Processes and Ecological Security in Changbai Mountains, Ministry of Education, School of Geographical Sciences, Northeast Normal University, Changchun, China; ^2^School of Ecology and Environment, Anhui Normal University, Wuhu, China; ^3^State Environmental Protection Key Laboratory of Wetland Ecology and Vegetation Restoration, Institute for Peat and Mire Research, Northeast Normal University, Changchun, China; ^4^Institute of Scientific and Technical Information of Jilin, Changchun, China

**Keywords:** Changbai Mountain, peatland restoration, soil enzyme activity, soil bacteria, soil properties, water regime

## Abstract

Appropriate hydrological management is critical for peatland restoration. An important prerequisite for peatland restoration is a recovery of soil biological processes. However, little is known about the effects of different hydrological management practices on soil biological processes during peatland restoration. In this study, the variations in soil properties, enzyme activities, and bacterial communities across different peatlands, namely natural peatland (NP), peatland restored under high water level (HR), peatland restored under alternating high-low water level (HLR), peatland restored under low water level (LR), and degraded peatland (DP), in the Changbai Mountains were investigated. Results showed that soil organic carbon, soil water content, and total nitrogen in NP were significantly higher than those in restored and degraded peatlands, and these soil properties in restored peatlands increased with the water level. The activities of soil hydrolases including β-1, 4-glucosidase, β-1, 4-n-acetylglucosidase, and acid phosphatase in NP were higher than in restored and degraded peatlands, while the activity of polyphenol oxidase in NP was the lowest. In restored peatlands, all measured enzyme activities decreased with the decline in water level. Both bacterial diversity and richness in NP were the lowest, while the highest diversity and richness were observed in HR. Redundancy analysis indicated that soil organic carbon, water level, soil water content, total nitrogen, and pH were the most important factors that affected the soil enzyme activities and bacterial community. Our findings give insight into the effects of different hydrological regimes on soil biological processes during peatland restoration. Maintaining a high water level early in the restoration process is more beneficial to restoring the ecological functions of peatlands than other hydrological regimes.

## Introduction

Boreal peatlands are important ecosystems because they store approximately one-third of the planet’s terrestrial carbon (Yu, 2012; Turetsky et al., 2015). However, human activities pose a major threat to peatland stability and cause various degrees of damage to natural peatlands (Dohong et al., 2017). Due to human disturbance, approximately 12.5% of the world’s peatlands have been lost or degraded (Frolking et al., 2011). The primary threat to peatlands is agricultural cultivation, which can destroy the native vegetation and hydrological regimes (Hallema et al., 2015). With the removal of native vegetation and the decline of the water level, organic matter decomposed quickly under aerobic conditions and soil carbon storage decreased (Berglund and Berglund, 2010; Heller and Zeitz, 2012; Hallema et al., 2015). These changes have shifted the world’s peatlands from a sink to a source of carbon (Kløve et al., 2010; Leifeld et al., 2019), which may have profound effects on global climate change. Therefore, it is urgent to develop suitable and sustainable restoration methods to restore degraded peatlands.

Peatland restoration measures mainly include plant reintroduction, hydrological mediation, ditch blocking, and alteration of microtopography (Peacock et al., 2015; Guo et al., 2016). The main purpose of peatland restoration is to restore ecological functions close to or to their undisturbed state by restoring hydrological conditions and plant communities (Lazcano et al., 2018; Ahmad et al., 2020). Planting has been considered to be an effective way to restore the dominant peatland species in degraded peatland, but vegetation alone cannot ensure the persistence of the restoration (Guo et al., 2016). In addition to vegetation, the primary challenge associated with restoration is hydrological restoration (Ahmad et al., 2020). Hydrological regimes strongly control the form and function of peatlands, because the water flow, dissolved minerals, and nutrients regulate the diversity and characteristics of the plant community (Belyea and Baird, 2006; Mitsch and Gosselink, 2007), as well as the production and decomposition dynamics that lead to the accumulation of peat (Moore et al., 2002). Therefore, a key consideration in peatland restoration is the management of the hydrological regime, which aims not only at the reestablishment of the original peatland vegetation but also at the rapid recovery of ecological functions.

Soil microorganisms play a key role in the biogeochemical functions of soils, such as soil organic matter formation, decomposition, and nutrient cycling, which further affect the carbon balance (Hill et al., 2014; Soares and Rousk, 2019; Qin et al., 2021). Soil biochemical properties, including microbial community structure and enzymatic activities, reacted quickly to alterations in soil physicochemical properties and water regimes (Sardans et al., 2008; Lagomarsino et al., 2009; Ma et al., 2020). Therefore, these soil biochemical properties were used as sensitive indicators of soil functions (Veres et al., 2015; Qin et al., 2021). Some studies have demonstrated that soil microbial communities and extracellular enzyme activities responded sensitively and drastically to peatland drainage, reclamation, grazing, and mining (Freeman et al., 1996; Ward et al., 2007; Burns et al., 2013). Peatland restoration, through the reestablishment of the original plant community or recovery of the hydrological regime, is always accompanied by increasing soil carbon, nitrogen, and soil water content (Lucchese et al., 2010; Putkinen et al., 2018), all these changes are likely to affect biological processes that drive soil functions (Purre et al., 2019; Ahmad et al., 2020). However, few studies have investigated the effects of peatland recovery under different hydrological management practices on soil biological processes.

The Changbai Mountain is the largest peatlands region in northeast China. The area of peatlands in this region is approximately 463.31 km^2^ (Ma et al., 2013). Since the 1950s, large areas of peatlands in this region have been cultivated into paddy fields after soil amendment. The area of peatlands was greatly reduced with the original hydrological patterns being destroyed (Li, 2013; Wang et al., 2020; Xu et al., 2021). Recently, the Chinese government issued the National Wetland Protection Law, which call for the restoration of the peatlands in China according to their types and degradation status. The Jilin Provincial Government developed and implemented plans for peatland restoration and intends to restore >6,000 hm^2^ of cultivated peatland in the Changbai Mountain. In this study, different hydrological management practices were implemented in the restored peatlands in the Changbai Mountain. The purposes of this study were (1) to reveal how soil microbial community structure and enzyme activities respond to different hydrological management practices, and (2) to explore the optimal hydrological management measure to restore the ecological functions of degraded peatlands.

## Materials and methods

### Study site

The study site is located at the west foot of the Changbai Mountain, approximately 1 km to the north of Sipeng Town, Tonghua City (41.858°N, 125.580°E), with an altitude of 512 m. The study area has a temperate continental monsoon climate. The mean annual precipitation and mean annual temperature are 790 mm and 5°C, respectively. The weather is usually cold and damp with about 110–150 frost-free days in a year (Song et al., 2005).

Before the 1980s, the study site was a typical peatland with a peat thickness of 0.8–1.2 m. The peatland area was approximately 40 hm^2^, and the dominant species was *Carex schmidtii*. In the early 2000s, about 90% of these peatlands were reclaimed as paddy fields, dry cropland, or fishponds. Then, in 2010, some paddy fields were abandoned under the call of the local government to protect wetlands. However, due to long-term agricultural cultivation, the native vegetation, biodiversity, topsoil peat layer, and hydrological regimes of the original peatland have been destroyed, and ecological services have declined dramatically (Planas-Clarke et al., 2020; Wang et al., 2020). In the abandoned paddy fields, although the peat layer still existed 30 cm below the surface soil layer, the vegetation were dominated by *Juncus bufonius*, *Echinochloa crusgali*, and *Bidens pilosa*, which are not typical peatland plants. The water level was significantly lower than that of the original peatland.

### Experiments and sample collection

**FIGURE 1:**
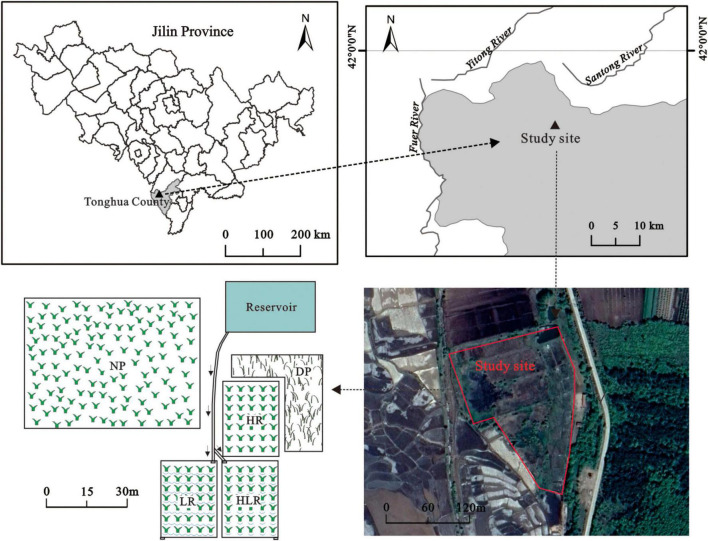
Study site locations in the Jilin province, northeastern China. NP, natural peatland; HR, peatland restored under high water level; HLR, peatland restored under an alternating high-low water level; LR, peatland restored under low water level; DP, degraded peatland.

In April 2019, to simulate different restoration measures, experiments were conducted on abandoned paddy fields that had been converted from natural peatland around 2,000 and were abandoned in 2010. The area of the paddy fields is approximately 0.5 hm^2^. There was a reservoir and a natural peatland near the paddy fields. The water of the reservoir came from rainfall and some nearby underground springs. The reservoir provides the water needed by the natural peatland, as well as water to conduct hydrological management in different experimental treatments (Figure 1). A natural peatland (NP), with an area of approximately 1.8 hm^2^, was taken as a control to evaluate the effectiveness of different restoration measures. The natural peatland has never been cultivated. The main vegetation community in the natural peatland is *C. schmidtii* and the peat thickness is 0.8–1.2 m. Before experiments, the abandoned paddy fields were under the same original hydrology and management. Environmental variables of the natural peatland and abandoned paddy fields are shown in Table 1.

**TABLE 1:** Environmental variables of the natural peatland and abandoned paddy fields before experiments.

	Soil organic carbon (g/kg)	Total nitrogen (g/kg)	Total phosphorus (g/kg)	pH	Soil water content (%)	Water level (cm)
Natural peatland	318.63 ± 12.53	21.77 ± 1.23	1.35 ± 0.14	5.46 ± 0.47	445.00 ± 23.14	1.37 ± 0.22
Abandoned paddy field	54.85 ± 4.37	4.39 ± 0.46	1.47 ± 0.11	6.12 ± 0.33	55.87 ± 5.67	−10.24 ± 1.06

The data in each column are shown as mean ± SE (n = 4).

To study the restoration process of peatland under different water regimes, the paddy fields were divided into four plots, with each plot (>700 m^2^) represent one treatment. Then, different restoration measures were implemented in the four plots, namely: (1) peatland restored under high water level (HR), the plot was permanently flooded through water supplement, and the water level was kept at a relatively high level: between 5 and 10 cm on average, (2) peatland restored under low water level (LR), the plot was under a relatively drained hydrological regime with a relatively low water level: among −10–0 cm on average, (3) peatland restored under an alternating high-low water level (HLR), the plot was under alternating flooded-drained hydrological regime with the water level alternating between high and low semimonthly, and (4) degraded peatland (DP), the plot maintained the status of abandoned paddy field with no restoration measures being conducted (Figure 1). At the beginning of the experiment, the three restored treatments (HR, LR, and HLR) were harrowed and then transplanted with *C*. *schmidtii*, with vegetation coverage of approximately 30–50%. After plant colonization, the restored peatlands were implemented with different hydrological management practices, hydrological management was carried out from May to October each year. The mean water levels of the restored peatlands during the growing season are shown in Table 2.

**TABLE 2:** Environmental variables in natural, restored, and degraded peatlands.

Environment variables	NP	HR	HLR	LR	DP
Soil organic carbon (g/kg)	325.00 ± 7.51a	73.23 ± 4.48b	60.65 ± 2.86bc	57.86 ± 3.10c	61.16 ± 2.40bc
Total nitrogen (g/kg)	24.90 ± 0.97a	5.57 ± 0.26b	5.47 ± 0.47b	4.86 ± 0.29b	5.62 ± 0.15b
Total phosphorus (g/kg)	1.41 ± 0.08d	1.88 ± 0.05a	1.75 ± 0.02ab	1.61 ± 0.04bc	1.58 ± 0.07c
Soil water content (%)	432.65 ± 70.37a	122.65 ± 40.35b	110.68 ± 6.35b	98.19 ± 4.74b	87.67 ± 3.47b
pH	5.56 ± 0.06bc	5.72 ± 0.15b	5.43 ± 0.56c	6.10 ± 0.04a	6.14 ± 0.02a
C/N	13.08 ± 0.29a	13.31 ± 1.36a	11.26 ± 0.78ab	11.92 ± 0.08ab	10.87 ± 0.15b
Water level (cm)	2.28 ± 0.44b	7.56 ± 0.17a	1.45 ± 0.10b	−6.89 ± 0.49c	−7.85 ± 0.26c

The data in each column are shown as mean ± SE (n = 4). The different letters behind the data indicate significant differences among different treatments (p < 0.05, LSD test). C/N, the ratio of SOC to TN; NP, natural peatland; HR, peatland restored under high water level; HLR, peatland restored under an alternating high-low water level; LR, peatland restored under low water level; DP, degraded peatland.

In October 2020, four 1 m^2^ sample plots were randomly selected from each treatment for soil sampling. At each sample plot, soil samples were collected from 4 points and mixed into a composite sample. The soil at the depth of 0–20 cm was collected using a soil borer with a diameter of 5 cm. Each soil sample was divided into two parts in the lab: one part was kept at −80°C for DNA extraction; the other part was kept at 4°C for soil enzyme activity and physicochemical analysis.

### Analysis of soil properties

The water level in different treatments was recorded using an Odyssey Logger (Dataflow Systems, Christchurch, New Zealand) installed in a PVC pipe. Soil pH was measured using a glass electrode (PHS-3E meter with E-201-C electrode, Leici, China). Soil water content was determined gravimetrically by drying at 105°C to a constant weight and then calculating the mass ratio of the water to the dried soil. Soil organic carbon (SOC) was determined after wet digestion with K_2_Cr_2_O_7_-H_2_SO_4_ and titration with FeSO_4_. The levels of total phosphorus and total nitrogen were determined by the Molybdenum blue method and the Kjeldahl method, respectively (Lu, 1999).

### Analysis of soil enzyme activities

The activities of three soil hydrolases and one oxidase: β-1, 4-glucosidase (βG), β-1, 4-n-acetylglucosidase (NAG), acid phosphatase (AP), and polyphenol oxidase (PPO), were determined by microplate fluorescence method (Saiya-Cork et al., 2002) using a multi-plate reader (Synergy H4 Hybrid Reader, Synergy H4BioTek, United States). The substrates used for the βG, NAG, AP, and PPO were 4-methyl umbelliferyl-BD-glucopyranoside, 4methyl umbelliferyl-BD-glucopyra-noate, 4-methyl parumone phosphate, and 4-dihydroxyphenylalanine, respectively. In brief, soil suspension was prepared by mixing about 0.5 g fresh soil sample and 125 mL sodium acetate buffer (pH = 5, 50 mmoL/L). To determine the activities of three soil hydrolases, 200 μL soil suspensions and 50 μL substrates were incubated in a 96-well microplate. The microplates were placed in the dark at 20°C for 4 h, and 10 μL of 1 mol/L NaOH solution was added and measured by the fluorescence detection method. To determine the activity of PPO, 600 μL soil suspension and 150 μL substrates were added to a transparent plate with a shallow mouth and incubated in the dark at 20°C for 5 h. Then the incubated solution was centrifuged under 3,000 r/min for 5 min and 250 μL supernate was sampled and measured using a microplate reader.

### Soil DNA extraction and high-throughput sequencing

Soil DNA was extracted from 1 g of soil from each sample using a Fast DNA™ SPIN Kit (MP Biomedicals, CA, United States). The concentration and purity of DNA extractives were examined and sent to Shanghai Majorbio Technology Co., Ltd., China, for high-throughput sequencing. The primer pairs used to amplify the V3-V4 hypervariable regions of the bacterial 16S rRNA gene were 338F (5′-ACTCCTACGGGAGGCAGCA-3′) and 806R (5′-GGACTACHVGGGTWTCTAAT-3′). An Illumina MiSeq platform was used for paired-end sequencing. The sequencing length of the target fragment was 250–500 bp.

The raw sequences were primarily processed with the standard Illumina pipeline. In brief, the raw sequences that passed through the mass screening were merged using Flash software (v1.2.11)^1^ to obtain raw tags (Magoč and Salzberg, 2011). Then, the raw tags were filtered using QIIME (v1.9.1)^2^ to identify the query sequences, and USEARCH (v7.0)^3^ was invoked *via* QIIME to examine and eliminate chimeric sequences, obtaining effective tags (Caporaso et al., 2010; Edgar et al., 2011). The effective tags were clustered into operational taxonomic units (OTUs) based on a sequence similarity threshold of 97% using the UPARSE pipeline (v7.0.1090)^4^ (Edgar, 2013), and each OTU was represented by the most abundant sequence in the OTU. An OTU table was created with the number of sequences of each OTU in each sample. Taxonomy was assigned for each OTU using the RDP Classifier (v2.11)^5^ based on SILVA (v138)^6^ (Quast et al., 2013). The low-abundance OTUs with a combined abundance of less than 10 sequences across all samples were eliminated.

### Statistical analysis

The Shannon and ACE indices were calculated for each sample using Mothur (v 1.30.2)^7^ (Schloss et al., 2009). One-way ANOVA followed by multiple comparisons using the LSD test was conducted to study the difference in microbial richness and diversity, soil properties, and enzyme activities among treatments. The significance level of *p* < 0.05 was considered asstatistically significant.

To study the effects of peatland restoration on soil enzyme activities and bacterial community composition, principal coordinate analyses (PCoA) were used on the Bray-Curtis dissimilarity matrices of the corresponding data. An ANOSIM test was used to verify the significance of the effects of hydrological regimes on bacterial community structure. Redundancy analysis (RDA) was used to explore the relationship between environmental variables and soil enzyme activities as well as microbial communities. Environmental variables were log-transformed and centered to equalize the weight of variables with ranges of different orders of magnitude. All data analyses were performed with R (v4.1.3)^8^ in R Studio (v 1.0.153)^9^ (Racine, 2012), the online platform of Majorbio Cloud Platform^10^ (Ren et al., 2022), and the SPSS 26.0 as needed.

## Results

### Environmental variables among different peatlands

Environmental properties differed significantly among different peatlands (Table 2). The highest SOC, total nitrogen, and soil water content were observed in NP, which had the lowest total phosphorus (*p* < 0.05). DP was characterized by the lowest water level, soil water content, and the ratio of soil carbon to nitrogen (C/N), and the highest pH (*p* < 0.05).

For restored peatlands, SOC and total phosphorus in HR were higher than that in LR, while pH in HR was lower than in LR (*p* < 0.05). Total nitrogen, total phosphorus, SOC, and soil water content decreased along with water level from HR to HLR to LR. No significant difference was found in total nitrogen, soil water content, and C/N among the three restored peatlands (*p* > 0.05). Furthermore, no significant difference was found in all the detected variables between LR and DP.

### Soil enzyme activities among different peatlands

**FIGURE 2:**
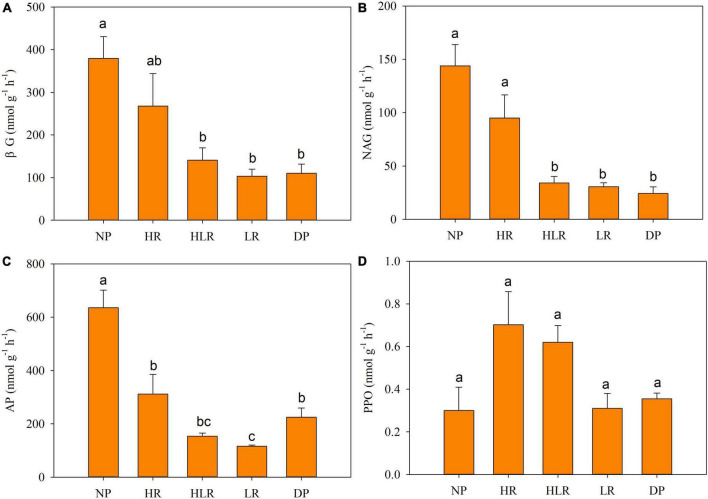
Soil enzyme activities in different peatlands. Bars represent average with standard error (*n* = 4). Different letters above the column indicate significant differences among different treatments, based on one-way ANOVA and LSD test (*p* < 0.05). βG, β-1, 4-glucosidase **(A)**; NAG, β-1, 4-n-acetylglucosidase **(B)**; AP, acid phosphatase **(C)**; PPO, polyphenol oxidase **(D)**; NP, natural peatland; HR, peatland restored under high water level; HLR, peatland restored under an alternating high-low water level; LR, peatland restored under low water level; DP, degraded peatland.

The NP was characterized by higher soil hydrolase activities and lower oxidase activities (Figure 2). The activities of βG, NAG, and AP in NP were significantly higher than those in other treatments (*p* < 0.05). The lowest activities of the four detected enzymes were in the LR or DP. The AP activity of LR was significantly lower than that of DP, no significant differences were found between DP and LR for the other three enzymes. In restored peatlands, the activities of all detected soil enzymes decreased along with decreasing water level from HR to HLR to LR.

**FIGURE 3:**
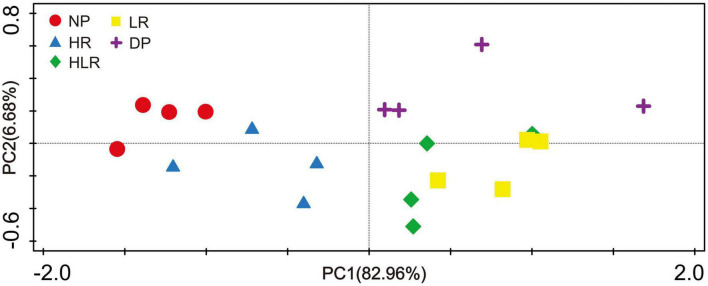
Principal coordinate analysis of soil enzyme activities across different peatlands. NP, natural peatland; HR, peatland restored under high water level; HLR, peatland restored under an alternating high-low water level; LR, peatland restored under low water level; DP, degraded peatland.

The PCoA showed that soil enzyme activities were significantly different across different peatlands. The first and second axes explained 82.96 and 6.68% of the total variation, respectively (Figure 3). All the points of NP and HR were present in the left quadrant, while all the points representing HLR, LR, and DP treatments were present in the right quadrant (Figure 3). In addition, the HR points were close to NP points, while most HLR and LR points were close to DP points.

### Soil bacterial community structure among different peatlands

**FIGURE 4:**
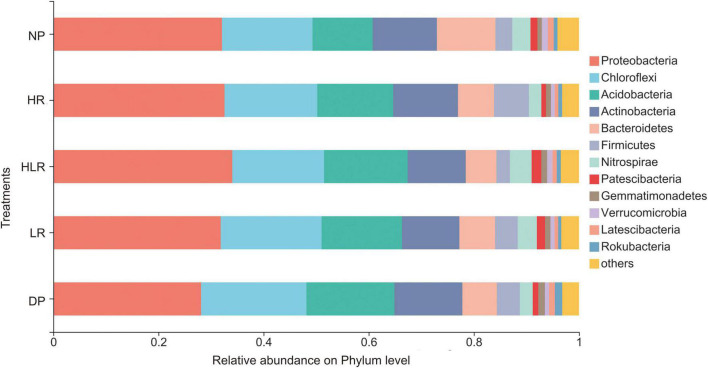
Relative abundance of different bacterial phyla under different peatlands. Only the top 12 phyla were displayed. NP, natural peatland; HR, peatland restored under high water level; HLR, peatland restored under an alternating high-low water level; LR, peatland restored under low water level; DP, degraded peatland.

In total, 5,540 bacterial OTUs with 7,74,127 sequences in all samples were obtained. These 5,540 OTUs were classified into 854 genera within 52 phyla. The largest phylum was Proteobacteria, followed by Chloroflexi, and Acidobacteria. The relative abundance of different bacterial groups changed markedly in different peatlands (Figure 4). NP was characterized by the highest relative abundance of Bacteroidetes and the lowest relative abundance of Acidobacteria. HR was characterized by a higher relative abundance of Firmicutes, while HLR had a higher relative abundance of Nitrospiraeand and Patescibacteria.

**FIGURE 5:**
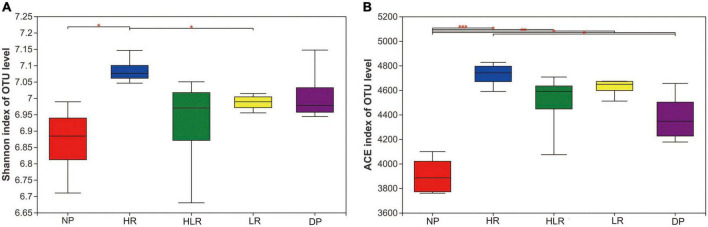
Shannon **(A)** and ACE **(B)** indices of soil bacteria from different peatlands. *, **, and *** mean *p* < 0.05, *p* < 0.01, and *p* < 0.001, respectively, based on one-way ANOVA and LSD test. NP, natural peatland; HR, peatland restored under high water level; HLR, peatland restored under an alternating high-low water level; LR, peatland restored under low water level; DP, degraded peatland.

The results of microbial alpha-diversity analysis indicated that HR is beneficial to the increase of bacterial diversity and richness (Figure 5). The Shannon index of HR was higher than NP and HLR (*p* < 0.05), and the ACE index of HR was higher than NP and LR (*p* < 0.05). The NP had the lowest Shannon and ACE indices among all treatments, with its ACE index lower than all other treatments, including DP (*p* < 0.05).

**FIGURE 6:**
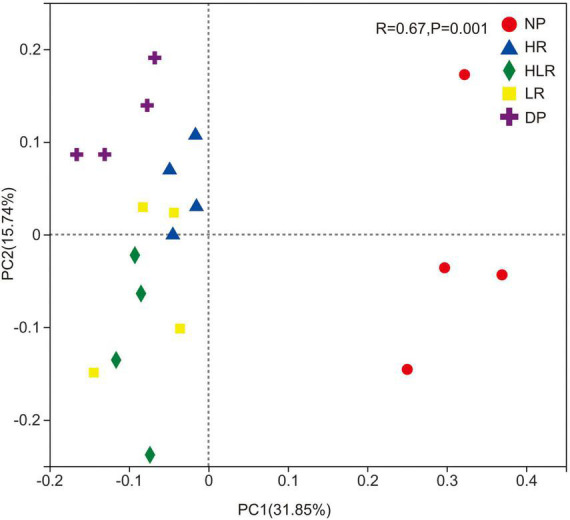
Principal coordinate analysis of the Bray-Curtis dissimilarity matrices of bacterial communities across different peatlands. The R and *p* were the statistical significance in bacterial community structure among different peatlands assessing by the ANOSIM test. NP, natural peatland; HR, peatland restored under high water level; HLR, peatland restored under an alternating high-low water level; LR, peatland restored under low water level; DP, degraded peatland.

The PCoA showed that bacterial community structure was significantly different across different peatlands. The first and second axes explained 31.85 and 15.74% of the total variation, respectively (Figure 6). Points representing bacterial communities of NP showed a clear separation from the points of the restored and degraded peatlands, indicating different community structures between natural peatland and the other treatments. Furthermore, the bacterial communities of restored peatlands were different from those of degraded peatlands, with their points showing a clear separation. For the restored peatlands, bacterial community structures showed similarities among the different treatments, shown as points clustered with each other in the plot.

### Effects of soil properties on enzyme activities and bacterial community

**FIGURE 7:**
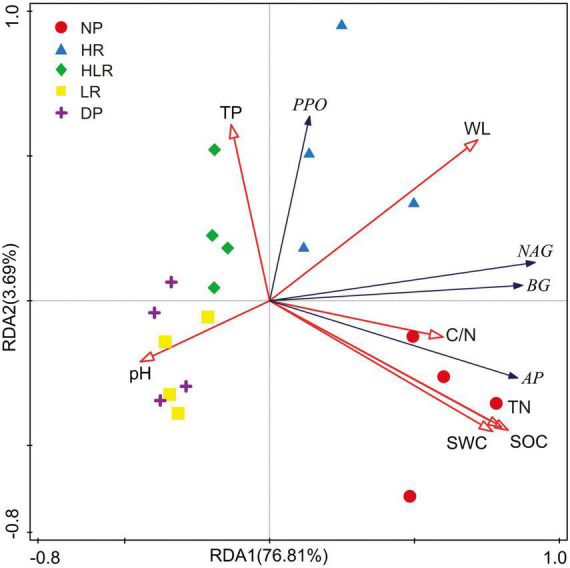
Redundancy analysis of soil enzyme activities across different peatlands using environmental variables as explanatory variables. βG, β-1, 4-glucosidase; NAG, β-1, 4-n-acetylglucosidase; AP, acid phosphatase; PPO, polyphenol oxidase; SOC, soil organic carbon; TN, total nitrogen; TP, total phosphorus; WL, water level; SWC, soil water content; C/N, the ratio of SOC to TN; NP, natural peatland; HR, peatland restored under high water level; HLR, peatland restored under an alternating high-low water level; LR, peatland restored under low water level; DP, degraded peatland.

The RDA indicated that soil enzyme activities were significantly influenced by environmental variables, with the first two axes explaining 80.5% of the total variations among different peatlands (Figure 7). Among these environmental variables, SOC explained the highest proportion of the variations in soil enzyme activities (51.0%, *p* = 0.002), followed by water level (21.2%, *p* = 0.002) and pH (4.3%, *p* = 0.044). The other measured soil properties, including soil water content, total phosphorus, total nitrogen, and C/N, explained only a small portion of the variations in soil enzyme activities, less than 2% in total.

**FIGURE 8:**
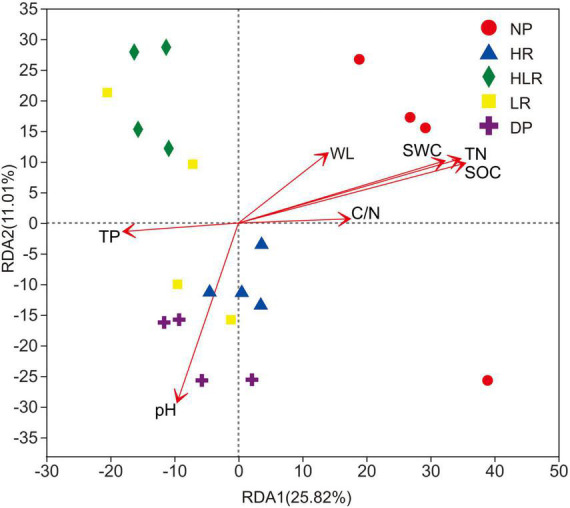
Redundancy analysis of the Bray-Curtis dissimilarity matrices of soil bacterial communities using environmental variables as explanatory variables. SOC, soil organic carbon; TN, total nitrogen; TP, total phosphorus; WL, water level; SWC, soil water content; C/N, the ratio of SOC to TN; NP, natural peatland; HR, peatland restored under high water level; HLR, peatland restored under an alternating high-low water level; LR, peatland restored under low water level; DP, degraded peatland.

The RDA showed that 50.53% of the total variations in the bacterial community were explained by environmental variables (Figure 8). Total nitrogen, SOC, soil water content, and pH were the most important factors affecting bacterial community. The bacterial community of DP was mainly affected by its higher pH, while the bacterial community of NP was mainly regulated by the higher SOC, total nitrogen, and soil water content.

## Discussion

### Responses of soil enzyme activities to different hydrological management practices

Soil enzyme activities play a key role in organic matter decomposition (Veres et al., 2015; Soares and Rousk, 2019). The detected soil enzyme activities of natural, restored, and degraded peatlands differed significantly (Figure 2), indicating that they were significantly affected by hydrological management practices during restoration. The hydrolysis enzyme activities (βG, NAG, and AP) in degraded peatland were significantly lower than those in natural peatland, this may be due to the sharp decrease in soil organic matter after peat reclamation. Previous studies also reported lower soil enzyme activities in agricultural or farm abandoned peatland than those in natural peatland (Wang et al., 2021). Peat cultivation can lead to rapid organic matter decomposition, and the nutrients released change to mobile forms that can be easily leached (Glina et al., 2016). These changes in degraded peatland result in a reduced carbon source, thus limiting soil enzyme activity (Hallema et al., 2015). However, the activity of polyphenol oxidase in NP was lower than other sites. This may be due to the high soil water content and poor soil air permeability in the natural peatland (Table 2), which is unfavorable to the survival and reproduction of aerobic microorganisms. As proposed by enzyme latch theory, under water logging conditions, a lack of oxygen can limit the activity of polyphenol oxidase (Freeman et al., 2001, 2004, 2012).

The soil enzyme activities of restored peatlands with high water level were higher than those of degraded peatland. Moreover, the soil enzyme activities in restored peatlands increased with water level, with enzyme activities being highest in HR and lowest in LR (Figure 2). Interestingly, the variations in enzyme activities in restored peatlands, unlike the condition in natural peatland, did not support the enzyme latch theory. A possible explanation is that flooding can hinder the decomposition of polyphenols in anoxic peat but not other organic matter (McGivern et al., 2021). For restored peatlands, after long-term rice cultivation and farm abandonment, the surface peat layer was completely destroyed, thoroughly changing the vegetation of restored peatlands. Therefore, most soil nutrients in restored peatlands were obtained from plants that were not typical peatland plants before peatland restoration, these nutrients were not hindered by the flooding environments. Instead, the higher soil enzyme activities in HR may benefit the accretion of organic matter, which benefited from the increasing plant biomass. The peatland was restored with *C. schmidtii* plantation, and *C. schmidtii* can easily adapt to the flooding environment with dense growth and multiply roots (Qi et al., 2021). In particular, the water depth of 11.2 cm, similar to the water level of HR in this study, promotes the rapid propagation of *C. schmidtii* and increases productivity to the maximum extent (Zhang et al., 2020). In addition, waterlogging in HR may have reduced the concentration of iron and decreased the protective effect on soil organic carbon (Wen et al., 2019). All these changes in plants and soil provided more available substrates for microorganisms and enhanced soil enzyme activities. The variations in soil properties also support this explanation.

### Responses of soil microbial communities to different hydrological management practices

The soil microbial community structure is a good indicator of changes in soil quality (Balser and Firestone, 2005). In this study, the bacterial community structure was significantly affected by different hydrological regimes during peatland restoration. Specifically, the bacterial community structure of HR was similar to that of NP, while LR was similar to DP. This was consistent with previous studies, which demonstrated that rewetting is an effective measure to restore peatland (He et al., 2015; Emsens et al., 2020). Moreover, our study further illustrates that rewetting with a higher water level was more effective than with a relatively lower water level.

Soil microbial diversity and richness can serve as indicators of ecosystem stability (Chaer et al., 2009). Previous studies indicated that degradation of peatlands leads to a decrease in diversity and richness of soil microorganisms, restored wetlands also showed lower microbial diversity and richness than natural wetlands (Xu et al., 2017; Kitson and Bell, 2020). However, in this study, the lowest bacterial diversity and richness were observed in NP among all peatlands, while the highest was found in HR, these results were unlike the variations in bacterial community structure. The same phenomenon was also observed in other studies (Andersen et al., 2013). This may be because the special environments of natural peatland, specifically, higher cellulose fraction and anaerobic environments, may restrict some microbes such as Acidobacteria. In this study, the relative abundance of Acidobacteria in natural peatland was lower than in restored and degraded peatlands, which was in line with the previous study (Emsens et al., 2020). A reasonable explanation for this is that some bacterial groups were restricted in natural peatland because they could not decompose recalcitrant organic matter such as cellulose and lignin, only being able to obtain nutrient substrates from other biology such as fungi that can decompose these recalcitrant organic matter (Boer et al., 2005; Jiao et al., 2022). The higher hydrolase activities but lower oxidase activities in the natural peatland also support this explanation. This further reinforces that the enzyme latch theory is effective only in natural peatlands, not restored peatlands, giving insight into the accumulative process of organic matter.

On the other hand, the higher diversity and richness of bacteria in peatland restored under high water levels may benefit from the combination of fresh organic matter derived from *C*. *schmidtii*, vascular plants, and some shrubs in abandoned rice paddies, which are thought to create favorable conditions for the development of active microbial biomass (Andersen et al., 2013). The RDA results indicated that SOC and total nitrogen were the most influential factors affecting the bacterial community, further supporting that bacteria were restricted by nutrient availability in natural peatland. Taken together, the environmental variables in natural peatland derived a different bacterial community that promotes organic matter accumulation but does not necessarily have a higher diversity and richness.

### Implication for peatland restoration

Against the background of growing agricultural needs, peatland drainage has become a common stress globally (Cris et al., 2014). Restoration is necessary to recover degraded peatlands (Kimmel and Mander, 2010; Lazcano et al., 2018). Generally, planting was conducted as the optimal strategy (Guo et al., 2016; Qi et al., 2021). However, we found no significant difference in soil enzyme activities and soil bacterial community structure between LR and DP. This result indicates that vegetation restoration alone, without recovery of the water regime, has difficulty restoring peatland ecosystem functions. In this study, among the three different hydrological management practices in the restored peatlands, the HR has the highest soil nutrients, soil enzyme activities, and soil microbial diversity and richness. The results of the current study along with previous studies indicate that rewetting with a relatively high water level can be an appropriate hydrological management measure to achieve carbon accumulation and biological activities during peatland restoration (Kimmel and Mander, 2010; Schulte et al., 2019; Ahmad et al., 2020). Our findings highlight hydrological management as an effective way to improve the soil nutrient cycling processes, promote the restoration of soil ecological function, and accelerate the restoration process of peatland. Our results also indicate that soil microbial properties are important biological indices that are sensitive to environmental changes and can be used as indicators to assess wetland restoration.

## Conclusion

In this study, we evaluated the effects of short-term peatland restoration in both hydrological management and original vegetation plantations on soil biological processes to support ongoing restoration efforts for the Changbai Mountain and other degraded peatland regions. Our results show that peatland restoration with a high water level (between 5 and 10 cm) can better promote the recovery of soil nutrients, enzyme activities, and bacterial communities compared to other hydrological management practices. This study gives insight into the mechanism underlying biological process changes affected by the water regime during peatland restoration and provides management strategies for northern peatland restoration.

## Data availability statement

The datasets presented in this study can be found in online repositories. The names of the repository/repositories and accession number(s) can be found below: https://www.ncbi.nlm.nih.gov/, PRJNA861699.

## Author contributions

MW and SW conceived and designed the experiments. CC and YW were responsible for the management of water level in the field. MW, SX, and LL analyzed the data and wrote the manuscript. All authors contributed to the article and approved the submitted version.

## Footnotes

1
https://ccb.jhu.edu/software/FLASH/

2
http://qiime.org/

3
http://www.drive5.com/usearch/

4
http://www.drive5.com/uparse/

5
https://sourceforge.net/projects/rdp-classifier/

6
https://www.arb-silva.de/

7
https://www.mothur.org/

8
https://www.r-project.org/

9
https://www.rstudio.com/

10
http://www.majorbio.com
